# Semi-quantitative *Influenza A* population averages from a multiplex respiratory viral panel (RVP): potential for reflecting target sequence changes affecting the assay

**DOI:** 10.1186/s12985-017-0796-3

**Published:** 2017-07-14

**Authors:** Kenneth H. Rand, Maura Pieretti, Rodney Arcenas, Stacy G. Beal, Herbert Houck, Emma Boslet, John A. Lednicky

**Affiliations:** 1 Department of Pathology, Immunology and Laboratory Medicine, University of Florida, Gainesville, FL 32610 USA; 2 BayCare HealthSystem, Clearwater, FL USA; 3 Roche Molecular System, Inc., Pleasanton, CA USA; 4 National Human Genome Research Institute, National Institutes of Health, Bethesda, MD USA; 5 Department of Environmental and Global Health, University of Florida, Gainesville, FL USA

**Keywords:** Influenza, Sequence drift, Rt Pcr, Multiplex viral PCR, Seasonal population average

## Abstract

**Background:**

Yearly influenza virus mutations potentially affect the performance of molecular assays, if nucleic acid changes involve the sequences in the assay. Because individual patient viral loads depend on variables such as duration of illness, specimen type, age, and immunosuppression, we examined seasonal population averages of positive tests to smooth inherent variability.

**Methods:**

We studied the population seasonal averages of the semi-quantitative nAMPs for the influenza matrix and hemagglutinin genes in the GenMark (Carlsbad, CA) Respiratory Viral Panel assay between 3 institutions over 3 Influenza seasons.

**Results:**

Population average nAMPs were strikingly consistent between separate institutions, but differed substantially between H3N2 and H1N1 seasons. In the 2012–2013 and 2014–2015 influenza seasons, matrix gene H3N2 nAMP averages were 50–70% less than those of the same assay in the 2013–2014 H1N1 season. Influenza strains representative of these seasons were grown in tissue culture and when the supernatant virus was adjusted to the same copy number using a TaqMan assay, the same relative differences were reproduced in the RVP assay. Because the sequences for the PCR and PCR product detection in the GenMark assay are proprietary, the manufacturer provided single stranded DNA matching the capture probe for the representative H3N2 (3 mismatches) and H1N1 strains (2 different mismatches). Equimolar concentrations of these synthetic DNA sequences gave average nAMP values that closely correlated with the average nAMPS of the representative strains and their respective seasonal averages.

**Conclusions:**

Seasonal averages of semi-quantitative data may provide a means to follow assay performance as a reflection of the effects of molecular drift.

## Background

*Influenza A* virus undergoes yearly antigenic drift that affects seasonal vaccine effectiveness. Nucleotide sequence changes primarily in the *hemagglutinin* (HA) gene underlie these yearly changes, and are affected by population-wide immunologic selection [1–5]. Mutation rates have been estimated to occur with a frequency of 5.72 × 10^−3^ nucleotide substitutions per site per year not only in the HA gene, but also independently in the matrix gene with a similar frequency of 5.39 × 10^−3^ nucleotide substitutions per site per year [6, 7]. Although molecular methods to detect Influenza virus RNA have targeted conserved areas of the HA and matrix genes, these methods are inherently subject to decreased sensitivity over time, as mutations accumulate in the target sequences of the assay. Examples of reduced sensitivity in molecular assays have been reported due to mutations in the Influenza matrix gene in 2012 and 2013 by Yang et al. in Taiwan [7], as well as in molecular assays for *M. tuberculosis*, and enterovirus, *Respiratory Syncytial Virus*, Hepatitis B, and influenza viruses [8]. In this study, we present data suggesting that sequence differences in the Influenza matrix gene between strains of H3N2 and H1N1 account for differences in the GenMark Respiratory Viral Panel (RVP) matrix gene assay nanoamperes (nAMPs), and that these differences are reflected in the population average mean ± SDs of all positive matrix gene tests over the course of an entire season.

## Methods

Our objective was to determine whether averaging the semi-quantitative nanoamperes (nAMPS) obtained from the GenMark (Carlsbad, CA) Respiratory Viral Panel (RVP) for all positive results for an entire Influenza season would show meaningful differences due to Influenza sequence drift.

### Study design

#### Patients studied

De-identified nAMP data from all patients who had a respiratory virus panel (RVP) (GenMark Diagnostics, Inc. eSensor, Carlsbad, CA) performed between 2012 – February, 2015 at UFHealth Shands Hospital, Gainesville, FL, BayCare Health System, Clearwater, FL and Pathology Consultants of South Broward, Hollywood, FL. All patient sample types (nasopharyngeal (NP) swabs, nasal swabs, throat swabs, sputums, endotracheal suction, bronchoalveolar lavage (BAL), etc.) were included in the study since the intent was to use population averages as a means of controlling for differences in clinical and sample collection variables.

#### Nucleic acid extraction

At UFHealth Shands Hospital, 200 μl of patient sample in viral transport medium was extracted with the MagnaPure compact (Roche Diagnostics, Indianapolis, IN), and was eluted in 50 μl of which 5 μl was added to the GenMark RVP assay. At Memorial Healthcare System, respiratory virus samples were extracted utilizing the easyMag (bioMerieux, Durham, NC). Two-hundred microliters of sample (nasopharyngeal swab) collected and transported in viral transport medium was prepared using the on-board protocol and extracted as per manufacturer recommendations. Nasopharyngeal swab samples not placed in viral transport medium were occasionally collected in Liquid Stuarts media. These samples were processed in 2 ml of lysis buffer (bioMerieux, Durham, NC) and prepared in an off-board extraction protocol as per manufacturer recommendations before loading onto the easyMag extractor. Nucleic acid was eluted in 60 ul with 5 ul being used in the GenMark RVP assay. At BayCare Health System 200 μl of patient sample was extracted using the QIAGEN QIAamp MinElute Virus Spin Kit on the semi-automated QiaCube system with on-board lysis. Nucleic acid was eluted in 60 μl and 5 μl were used in the GenMark RVP assay.

#### GenMark RVP assay

The GenMark RVP panel was used according to instructions from the manufacturer. The extracted nucleic acid is reverse transcribed and amplified using viral specific primers with RT-PCR enzyme mix. The amplified DNA is converted to single-stranded DNA via exonuclease digestion and is then combined with a signal buffer containing ferrocene-labeled signal probes that are specific for the different viral targets [9]. A visual description is available at https://www.genmarkdx.com/solutions/technology/esensor/. Amplicon detection is measured by the peak height of a current flowing between the gold electrode and the ferrocene labeled probe, which is brought into proximity with the electrode by the capture probe binding to the target amplicon. Thus the peak current flow in nanoAMPs is a function of the number of targets bound to the capture probes and the tightness of this bond. The technology is sufficiently sensitive to be able to detect single basepair(bp) mutations in cystic fibrosis (https://genmarkdx.com/solutions/panels/xt-8-panels/cystic-fibrosis-genotyping-test/) and in thrombophilia (https://genmarkdx.com/solutions/panels/xt-8-panels/thrombophilia-risk-test/).

#### TaqMan assay

We developed a TaqMan PCR for conserved regions of the matrix gene that had no sequence variations in the primers and probe when matched against an environmental H1N1 strain from 2013 to 2014 season isolated at the University of Florida by one of the authors (JL) (*Influenza* A virus (A/environment/Gainesville/01/2014(H1N1) KJ 195790), and against sequences of A/New York/39/2012 (H3N2) FR-1307, A/Texas/50/2012 (H3N2) FR-1210, and A/Switzerland/9715293/2013 (H3N2) FR-1368 obtained from IRR/ATCC. The sequences for the assay are as follows:

- Forward primer 89-S: CCG AGA TCG CGC AGA GAC.
- Reverse primer 239-AS: GCT CAC TGG GCA CGG TG.
- Probe 177-AS-Pr: ATT GGT CTT GTC TTT AGC CAT TCC ATG AGA G.

The TaqMan assay was used to match viral RNA copy number for supernatant tissue culture fluid for these 4 strains grown in tissue culture. Tissue culture fluids were extracted, adjusted appropriately for differences in concentration as measured by the TaqMan assay and run in the RVP in quadruplicate.

#### Influenza matrix gene sequencing

The matrix gene for the 4 Influenza strains were sequenced by traditional Sanger sequencing in the UF Biotechnology Core laboratory facility, using the following primers for an approximately 800 base pair (bp) product:

- Forward primer 89‐S:CCG AGA TCG CGC AGA GAC
- Reverse sequencing primer ATATTC TTCCCT CAT RGA CTC AG

Since the RVP Influenza assay sequences are proprietary, sequences were sent to GenMark Diagnostics who provided data showing the number and location of mismatched base pairs in the Matrix and HA gene sequence in the RVP forward and reverse primers, capture probe and signal probe.

### Influenza HA gene sequences

The following Influenza HA gene sequences were obtained from GenBank and were sent to GenMark for matching with their subtype assay sequences: A/Texas/50/2012 (H3N2) KJ942616.1, A/Florida/15/2014(H3N2) KM064336.1, A/Florida/32/2014(H3N2) KR057571.1, A/Florida/21/2014(H3N2) KM972893.1, A/Florida/35/2014(H3N2)KT836943.1 and A/environment/Gainesville/01/2014(H1N1) KJ195788.

### Cell cultures

MDCK-SIAT2,6-UF cells [10, 11], which overexpress influenza virus α2,6-linked sialic acid receptors, were used for the isolation of influenza viruses. The cells were propagated as monolayers at 37 °C and 5% CO2 in Advanced Dulbecco’s Modified Eagle’s Medium (Invitrogen Corp., Carlsbad, CA, USA) supplemented with 2 mM L-Alanyl-L-Glutamine (GlutaMAX, Invitrogen Corp.), antibiotics (50 μg/mL penicillin, 50 μg/mL streptomycin, 100 μg/mL neomycin (Invitrogen Corp.)), and 10% (*v*/v) low IgG, heat-inactivated gamma-irradiated fetal bovine serum (HyClone, Logan, Utah).

### Viruses

*Influenza* virus A/environment/Gainesville/01/2014(H1N1) (GenBank KJ195790) was isolated from collection media used during air sampling of classroom air (J. Lednicky, unpublished). Viruses A/New York/39/2012 (H3N2), A/Texas/50/2012 (H3N2), and A/Switzerland/9715293/2013 (H3N2) were obtained from the American Type Culture Collection Influenza Reagent Resource (catalog numbers FR-1307, FR-1210, and FR-1368).

### Virus isolation and propagation

For primary isolation or passage of stock viruses, aliquots of samples containing influenza virus were inoculated onto newly confluent MDCK-SIAT2,6-UF in serum-free aDMEM otherwise supplemented as described above plus L-1-tosylamido-2-phenylethyl chloromethyl ketone treated mycoplasma-and extraneous virus-free trypsin (Worthington Biochemical Company, Lakewood, NJ) in 5% CO2 at 33 °C. The TPCK-trypsin was used at a final concentration 2 μg/mL. For virus passage, cells were infected at a multiplicity of infection of 0.01 or less. The inoculated cells were monitored daily for influenza virus-specific cytopathic effects (formation of focal enlarged granular cells followed by sloughing in rapid progression); in most cases, about 80% of the virus-infected cells had detached from the growth surface at the end of 2 days of infection. The presence of *influenza* A virus in the cell culture media was quickly determined using a commercial solid phase ELISA test (QuickVue *influenza* A and B kit, Quidel Corp., San Diego, CA, USA), and the viral gene sequences determined after RT-PCR and sequencing as described previously [11].

#### Statistics

ANOVA and Chi-squared were performed online as referenced in Tables 1 and 2. T-tests were performed on line at http://www.graphpad.com/quickcalcs/ttest1.cfm.

**Table 1:** Influenza matrix and subtype gene seasonal nAMP averages in 3 institutions over 3 influenza seasons

	Influenza season			
	2012–2013 H3N2 nAMPs ± SD (n)	2013–2014 H1N1 nAMPs ± SD (n)	2014–2015 H3N2 nAMPs ± SD (n)	P
Matrix Gene				
UF Health	121.1 ± 109.5 (91)^1^	172.6 ± 70.2 (194)^2^	65.1 ± 37.5 (172)	< 0.0001^4^
BayCare	74.8 ± 77.8 (19)	165.4 ± 76.1 (232)	50.2 ± 25 (152)	< 0.0001^4^
Memorial	ND^3^	160.1 ± 80.6 (475)	57.3 ± 61.1 (788)	< 0.0001^4^
Subtype Gene				
UF Health	169.9 ± 63.6 (91)	240.9 ± 103.4 (194)	164.3 ± 40.1 (172)	< 0.0001^4^
BayCare	136.1 ± 50.2 (19)	249.2 ± 81 (239)	162.4 ± 45.9 (165)	< 0.0001^4^
Memorial	ND	239.4 ± 88.6 (475)	144.6 ± 53.0 (711)	< 0.0001^4^

^1^() = number of specimens tested

^2^Includes 22 patients from 1/21/2013–8/30/2013 who were positive for H1N1

^3^ND = Not Done

^4^ANOVA http://vassarstats.net/anova1u.html

**Table 2:** Ct adjusted nAMPs and GenMark RVP Matrix Gene Sequence Mismatches

Influenza Strain	Matrix Gene nAMPs^a^	Matrix Gene Mismatches			
		Forward	Reverse	Capture	Signal
A/NY	53.5 ± 32^b^	1^c^	0	3^d^	0
A/Texas	56.6 ± 11.4^b^	1	0	3	0
A/Switzerland	46 ± 16.9^b^	1	0	3	0
H3N2 Synthetic Capture Probe ssDNA^e^	73 ± 16	N/A^f^	N/A	3	N/A
H1N1	143.6 ± 57.2	1	0	2	0
H1N1 Synthetic Capture Probe ssDNA	151 ± 17	N/A	N/A	2	N/A

^a^Mean ± SD of 4 replicates. All 4 strains were diluted to the same copy number based on Ct using a TaqMan assay (see Methods)

^b^A/NY (*p* = 0.033), A/Texas (*p* = 0.025) and A/Switzerland (*p* = 0.017) vs H1N1 t test. https://www.usablestats.com/calcs/2samplet

^c^The mismatches were the same in all H3N2 strains, but different from the H1N1

^d^All 3 mismatches were the same for the H3N2 strains, but differed from the 2 mismatches in the H1N1 strain

^e^ssDNA = single stranded DNA

^f^N/A = not applicable

## Results

**Fig. 1:**
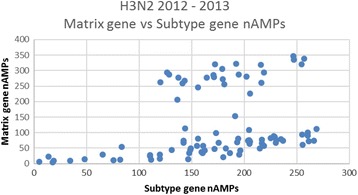
Matrix gene nAMPs graphed vs Subtype gene for 2012–2013 H3N2 season at UFHealth Shands hospital. There appears to be a subpopulation with relatively higher Matrix gene nAMPs

**Fig. 2:**
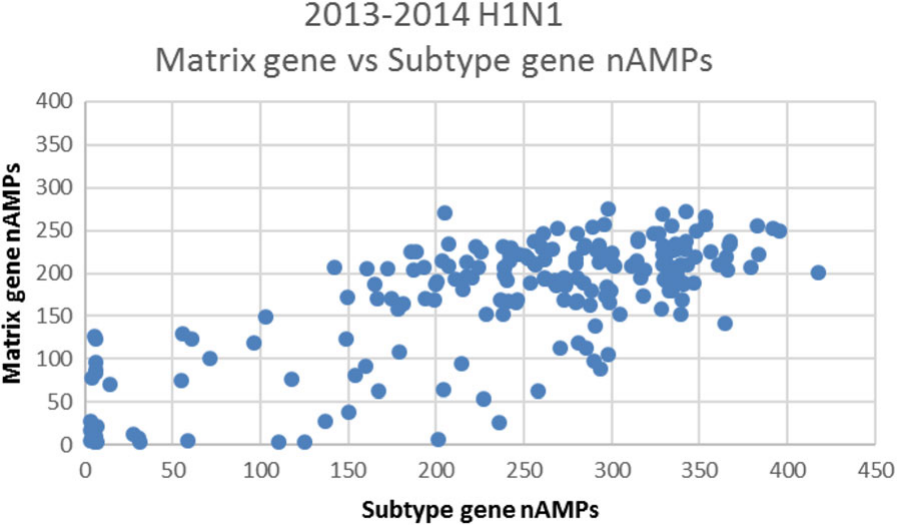
Matrix gene nAMPs graphed vs Subtype gene for 2013–2014 H1N1 season at UFHealth Shands hospital. There appears to be a relatively uniform ratio of the Matrix gene to Subtype gene nAMPs

**Fig. 3:**
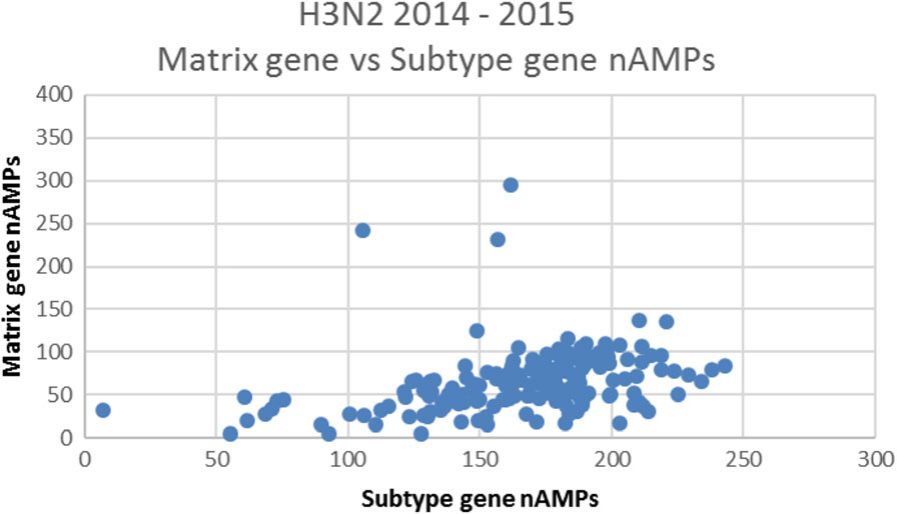
Matrix gene nAMPs graphed vs Subtype gene for 2014–2015 H3N2 season at UFHealth Shands hospital. In contrast to the 2012–2013 season, except for 3 individuals, there is an essentially uniform ratio of the Matrix gene to Subtype gene nAMPs

Population seasonal nAMP means ± SDs are shown in Table 1 and were consistent between institutions within a given year. For example, in Table 1 in 2014, nAMPs of 172.6 ± 70.2 (*N* = 194), 165.4 ± 76.1 (*N* = 232) and 160.1 ± 80.6 (*N* = 475) were calculated for the Matrix gene assay for all positive H1N1 strains at UFHealth, BayCare Health System, and Memorial Healthcare System respectively. These institutions are 150–300 miles apart and do not share patient populations. In contrast, mean ± SD matrix gene nAMPs for the 3 different Influenza seasons (2012–13, 2013–14, and 2014–15) were highly statistically different, the 2013–14 H1N1 seasonal average being more than twice that of the 2014–15 season (ANOVA, *p* < 0.0001). A similar pattern was observed for the HA subtype gene assay as well (ANOVA, *p* < 0.0001). The 2012–2013 H3N2 matrix gene nAMPs appear to be higher than those of the 2014–2015 H3N2 season and further analysis (see Figs. 1, 2, 3) suggests that in fact the average nAMPs in the 2012–2013 H3N2 season at UFHealth may have been bi-modal, whereas the distribution is clearly unimodal for 2013–2014 and 2014–2015. In these Figures, the matrix gene nAMPs are graphed vs the corresponding subtype gene nAMPS, making the subpopulation in the 2012–2013 season easily visualized.

When adjusted for the number of input RNA copies based on the cycle-threshold (Ct) of the TaqMan matrix gene assay, H3N2 strains representative of those circulating in the 2012–2013 and 2014–2015 seasons had significantly lower average nAMPs than the H1N1 strain from 2013 to 2014 (see Table 2) and these average nAMPS were very close to those of the seasonal population averages. As was the case for the population averages, the average nAMPS of the TaqMan copy number adjusted H3N2 strains were statistically significantly lower than that of the H1N1 strain (*p* = 0.033 vs A/NY, *p* = 0.025 vs A/Texas and *p* = 0.017 vs A/Switzerland, t-test).

Matrix gene sequencing of A/New York/39/2012 (H3N2) FR-1307, A/Texas/50/2012 (H3N2) FR-1210, A/Switzerland/9715293/2013 (H3N2) FR-1368 and the Gainesville environmental 2013–2014 isolate A/environment/Gainesville/01/2014(H1N1) KJ195788 showed the same 3 bp mismatches in the capture probe for the H3N2 strains as opposed to only 2 different ones for the H1N1 when compared with the sequence of the proprietary GenMark RVP assay. Discussion of the location of these mismatches with GenMark suggested the 3 mismatches in the H3N2 strains were likely to be more destabilizing than the 2 mismatches in the H1N1 strain. Please see Table 2 for complete details. Since the GenMark assay sequence details are proprietary, specific sequence data as to which mutations were observed is not available. In contrast, the subtype genes of both the H3N2 and H1N1 strains have a perfect sequence match with their respective subtype capture and signal probes and a single bp mismatch in both forward and reverse primers.

In order to confirm and better understand the relationship between sequence differences and nAMPs, GenMark Diagnostics provided single-stranded DNA matching the capture probe sequences of the H3N2 and H1N1 strains. When this synthetic DNA was run in the eSensor, equimolar concentrations gave average nAMPs of 150.5 ± 17.2 and 72.9 ± 16.3 nAMPs (*N* = 4, *p* = 0.0006, t-test), for the sequences matching the H1N1 strain and H3N2 strains, respectively (see Table 2). A capture probe with a perfect sequence match to the GenMark assay was tested at GenMark and gave the same average nAMPs as the H1N1 sequence, despite the 2 mismatches in the H1N1 sequence (data not shown).

## Discussion

The use of respiratory virus quantitation has been discussed primarily in relation to severity of individual patient illness and duration of viral shedding [10–12]. Clearly such quantitative values, whether GenMark nAMPs or TaqMan Ct values from commercial or lab developed assays, vary greatly from patient to patient and even within a patient depending primarily time of sample collection since days 1 and 2 of illness typically have the highest titers [12–14]. Other sources of quantitative difference result from variability in specimen type (e.g. nasopharyngeal vs BAL), variation in collection practices within the same specimen type, as well as patient variables such as immunosuppression, prior vaccination, age, etc. Although the semi-quantitative nAMP readings are not FDA approved for use in patient care, we looked at viral quantitation from the perspective of population seasonal averages, so that patient-related and pre-analytic variability should average out. Our data suggest that this is the case. Seasonal *Influenza* A population matrix gene nAMPs from the GenMark RVP assay were remarkably consistent between 3 different institutions in Florida for 3 Influenza seasons from 2012 to 2015. The slightly higher matrix gene nAMPs seen at UFHealth in 2012–2013 compared with 2014–2015 seems to be explained by a bi-modal distribution in the 2012–2013 season, suggesting there may have been a mixture of strains in circulation.

The 2013–2014 H1N1 season matrix gene nAMPS were markedly higher than the prior and following H3N2 seasons. Since the same matrix gene assay is used for both subtypes, it was possible these differences could have reflected seasonal differences in Influenza illness severity; however, sequence analysis suggested that the differences were better explained by sequence drift in the matrix gene that had occurred by the 2012–2013 season, and was unchanged in the 2014–2015 season. Since the nAMP averages from the synthetic single stranded DNA of the H3N2 and H1N1 capture probe sequences closely matches those of the seasonal averages, sequence variation between the H3N2 and H1N1 viruses offers the best explanation for the seasonal differences.

Further study is needed to determine whether population averages could reflect the clinical intensity of an influenza season, once it was determined that no assay-related sequence drift had occurred, on the theory that more severely ill the patients might seek medical care earlier in the course of their illness. Hence higher titers could be a reflection of patients being tested earlier when their titers were higher [12–16]. As noted above, the subtype genes of both the H3N2 and H1N1 strains have a perfect sequence match with their respective subtype capture and signal probes and a single bp mismatch in both forward and reverse primers, but since each subtype assay has different target sequences, average nAMPs cannot be compared.

One of the limitations of this study is that we did not save the actual strains from the 3 influenza seasons studied, since the laboratory was no longer performing viral cultures. However, the H3N2 strains analyzed for the matrix gene in the 2012–2013 and 2014–2015 seasons are generally considered representative of the sequence variation observed in the matrix genes, and the H1N1 isolate was an environmental isolate obtained on the University of Florida campus near the peak of the 2013–2014 season. Likewise, sequence analysis of HA sequences available from GenBank for representative H3N2 strains showed no changes in the number and location of mismatches in the GenMark assay between the 2012–2013 and 2014–2015 seasons. Another issue, although not strictly a limitation of the study, is that the GenMark assay is more complex than traditional TaqMan assays, in that after the PCR product is produced, it is captured on a solid phase where a 4th probe that carries a ferrocene label is required to bind to permit detection of an electrical signal. Although a positive assay is reported from 3.0 to >300 nAMPs, in our experience the actual linear range is only about 10–30 fold, which is very narrow compared with the generally observed 5 logs of linearity for a typical TaqMan assay.

Perhaps the most important implication of this study is that quantitative population averages (whether done by nAMPs as in the GenMark assay, or by Ct as would be the case in other manufacturers’ assays) appear to be sensitive to Influenza sequence changes due to seasonal drift that result in assay mismatches. In addition, it is possible that plotting the nAMPs of the matrix gene vs the subtype gene may offer a simple method to detect subpopulations with sequence variations affecting the performance of the assay. Although it does not appear that any Influenza strains were actually “missed” in our study because of mismatches between the matrix gene assay and the matrix gene sequence in circulating strains, further sequence drift in circulating strains could render the assay falsely negativee at some point.

## Conclusions

Seasonal average nAMPs were remarkably consistent between institutions within a given year. The differences in the matrix gene averages between H3N2 and H1N1 seasons were consistent with the number and location of mismatches in the molecular assay. Instrument manufacturers, laboratories and regulatory agencies should work out an approach to capture this type of data electronically on national and regional levels so that seasonal sequence drift affecting the performance of molecular assays can be monitored.

## Abbreviations

BAL: Bronchoalveolar lavage
bp: Base pair
Ct: Cycle threshold
HA: *Hemagglutinin*
nAMPs: Nanoamperes
NP: Nasopharyngeal
RT-PCR: Reverse transcriptase polymerase chain reaction
RVP: Multiplex respiratory viral panel
